# Evolutionary Trajectories of Shoots vs. Roots: Plant Volatile Metabolomes Are Richer but Less Structurally Diverse Belowground in the Tropical Tree Genus *Protium*

**DOI:** 10.3390/plants14020225

**Published:** 2025-01-15

**Authors:** Katherine D. Holmes, Paul V. A. Fine, Italo Mesones, Julieta Alvarez-Manjarrez, Andressa M. Venturini, Kabir G. Peay, Diego Salazar

**Affiliations:** 1 Department of Biological Sciences, Binghamton University, Binghamton, NY 13902, USA; dsalazaramor@binghamton.edu; 2 Biology Department, Florida International University, Miami, FL 33199, USA; 3 Department of Integrative Biology, University of California Berkeley, Berkeley, CA 94720, USA; paulfine@berkeley.edu (P.V.A.F.);; 4 Instituto de Biología, Universidad Nacional Autónoma de México, Mexico D.F. 04510, Mexico; julieta.alvarez@ib.unam.mx; 5 Department of Biology, Stanford University, Stanford, CA 94305, USA; aventuri@stanford.edu (A.M.V.); kpeay@stanford.edu (K.G.P.); 6 Department of Environmental Science, American University, Washington, DC 20016, USA

**Keywords:** chemical diversity, evolution, richness, plant–herbivore, rhizosphere

## Abstract

The breadth and depth of plant leaf metabolomes have been implicated in key interactions with plant enemies aboveground. In particular, divergence in plant species chemical composition—amongst neighbors, relatives, or both—is often suggested as a means of escape from insect herbivore enemies. Plants also experience strong pressure from enemies such as belowground pathogens; however, little work has been carried out to examine the evolutionary trajectories of species’ specialized chemistries in both roots and leaves. Here, we examine the GCMS detectable phytochemistry (for simplicity, hereafter referred to as specialized volatile metabolites) of the tropical tree genus *Protium*, testing the hypothesis that phenotypic divergence will be weaker belowground compared to aboveground due to more limited dispersal by enemies. We found that, after controlling for differences in chemical richness, roots expressed less structurally diverse compounds than leaves, despite having higher numbers of specialized volatile metabolites, and that species’ phylogenetic distance was only positively correlated with compound structural distance in roots, not leaves. Taken together, our results suggest that root specialized volatile metabolites exhibit significantly less phenotypic divergence than leaf specialized metabolites and may be under relaxed selection pressure from enemies belowground.

## 1. Introduction

Neighboring closely related plant species in the tropics often display greater divergence in chemical traits than expected by chance [1,2,3,4]. This pattern has been attributed to pressure from shared herbivores [5] that select hosts based on chemical composition [6]. Given that communities comprising plants with similar traits often attract larger populations of specialized insect herbivores [7], plant species should experience greater competitive advantage when they express distinct chemical profiles from their neighbors, enabling the coexistence of close relatives [8,9]. In combining perspectives from community ecology and evolutionary biology, we might expect phenotypic divergence in chemistry to be particularly high in lineages where the co-occurrence of close relatives is the norm—as is frequently the case in the tropics [2,5].

The expectation that shared enemies drive phenotypic divergence among co-occurring plants relies on a few assumptions. Two important assumptions here are (i) the relative ease of enemy dispersal and choice between potential hosts and (ii) at least moderate levels of host specialization. While this may frequently be the case in populations of aboveground insect herbivores moving between individual hosts or across different host populations in a manner of hours (e.g., [10,11]), plants encounter a different world of enemies belowground (e.g., nematodes, bacteria, and fungi [12]) that might not be as mobile or as specialized as aboveground plant natural enemies. Pathogenic microorganisms can show an extremely large variation in host range breadth, from being species-specific to colonizing thousands of plant hosts. Although a formal comparison of host specificity between herbivores and fungal pathogens has not yet been explored, insect herbivores seem to show a much higher degree of host specialization (as most species are considered specialists on a small number of closely related taxa, [13]) when compared to belowground fungal pathogens (where species are commonly specialized on larger plant clades and host ranges can change across environmental gradients, [14,15,16]). In terms of dispersal, fungal pathogens can show very large geographical ranges and, thus, very effective large-distance dispersal mechanisms [17,18,19]; nevertheless, many insect herbivores can move from host to host in a matter of minutes. In particular, the limited ability of root pathogenic fungi and bacteria to quickly disperse between nearby hosts populations compared to insect herbivores [20] may cause marked differences in selection on phenotypic divergence in chemical defenses against above- versus belowground enemies for sympatric, closely related plant species.

Research on foliar fungal pathogens suggests that extreme specialization appears to be rarer and to exert less pressure for chemical divergence relative to insect herbivory [3]. Nevertheless, the effectiveness of pathogens can depend on host phylogeny [21]. Plant roots are known to produce a wide variety of compounds that can grant protection against microbial pathogens. Defensive metabolites are frequently found in root exudates, both constitutively and in response to infection by fungi or bacterial pathogens [22,23]. While there can be broad overlaps between the classes of compounds produced by roots and leaves (e.g., phenolics and terpenoids), different compound classes often dominate each sphere [24]. For instance, in *Eucalpytus*, phenolic compounds are produced in comparable concentrations in leaves and roots, but terpenes have been detected in much lower amounts in roots [25]. This is not surprising, given the differences in the specialization and dispersal of below- and aboveground natural enemies. We can also expect to find significant differences in the diversity and composition of plant metabolites across above- and belowground plant tissues. Nevertheless, are these two selective environments creating fundamentally different evolutionary trajectories for plant-specialized phytochemicals?

Another key difference between the leaves and roots that could also influence their chemical evolutionary trajectories is the apparent paradox faced by root chemical defenses [26]. Plants must achieve a critical balance between the evolution and expression of chemical defenses against root bacterial and fungal pathogens while maintaining healthy symbiotic relationships with bacterial and fungal rhizosphere partners, most of which are also mediated and maintain by specialized metabolites [27,28].

Comparing the specialized metabolite diversity and composition of roots vs leaves across a plant clade may help evaluate differential evolutionary pressures across different plant tissues. Examining the richness and structural variation in compounds in leaves versus roots can also address hypotheses surrounding the origins and function of phytochemical diversity [29]. This includes the idea that the accumulation of novel chemical traits and corresponding divergence from close relatives and community members allows for enemy escape and that metabolomic richness counters the challenge of a diverse community of enemies [30]. Thus, it is generally expected that chemical diversity is likely to mirror natural enemy diversity.

To explore patterns of phenotypic divergence in above- versus belowground specialized chemistry, we investigated *Protium*, a species-rich genus of tropical trees that includes more than 100 Amazonian taxa, including 10 species considered “hyperdominant” in the Amazon basin [31]. The majority of *Protium* taxa originated in the last 10 million years, and the analysis of phylogenetic community structure suggests that numerous parapatric or allopatric speciation events occurred within different regions of the Amazon [32]. This has resulted in the broad co-occurrence of closely related *Protium* species across the Amazon basin, creating community dynamics where phenotypic divergence from close relatives may be particularly critical in the presence of common enemies [33]. To compare the composition of specialized metabolites above- versus belowground, we analyzed the leaf and root tissues of 31 *Protium* species that grow in the Allpahuayo–Mishana National Reserve near Iquitos, Peru.

Specifically, we analyzed the low-molecular-weight specialized metabolites of leaves and roots detectable via GCMS and compared their diversity, composition, and phylogenetic distribution across the *Protium* clade. Here, we also used a novel approach aimed at assessing the differences in structural alpha and beta diversity between root and leaf specialized metabolites independently of chemical abundance and richness. Given our expectation that the above- and belowground natural enemy communities were likely to significantly differ in their ability to disperse and track their plant hosts, and that root chemical defenses were likely to be more constrained to maintain positive symbiotic belowground interactions, we expected to see corresponding differences in the levels of divergence of specialized chemistry among close relatives in roots versus leaves.

## 2. Methods

To compare the evolutionary trajectories of specialized metabolites in roots vs leaves, we analyzed both previously collected chromatographic data on *Protium* leaf extracts and newly collected data on root extracts of the same species and field site.

### 2.1. Sample Collection

**Figure 1:**
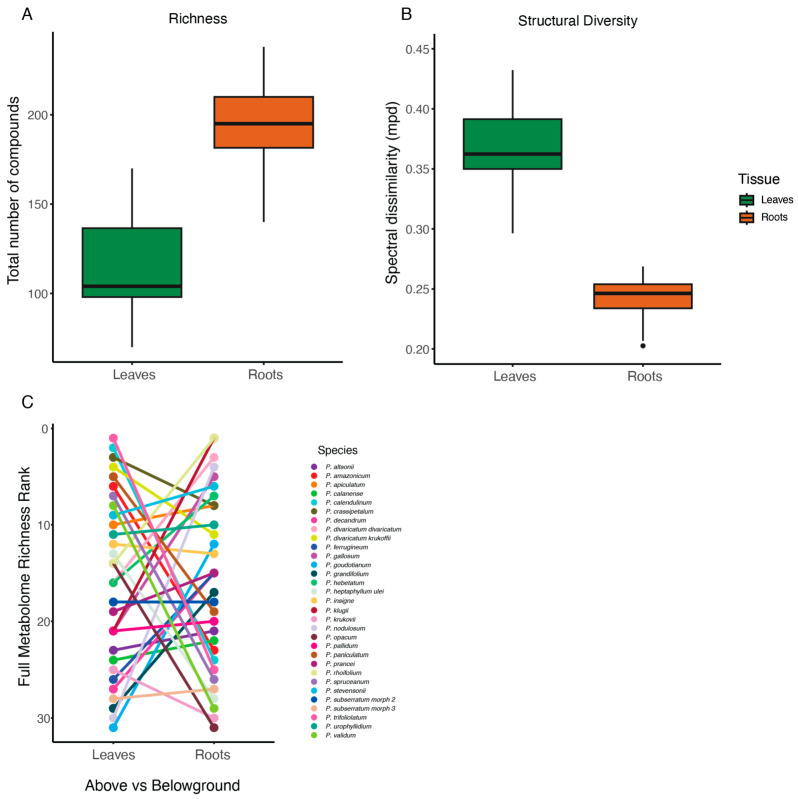
(**A**) We found significant differences in compound richness between roots and leaves across our 31 focus *Protium* species. (**B**) Differences in structural diversity between roots and leaves across our 31 experimental *Protium* species. (**C**) Even when ranking *Protium* species based on metabolomic richness to control for the effect of large differences between specific species pairs, there was no relationship between leaf and root richness at the species level.

Leaf and root samples were collected from plants located within the Allpahuayo–Mishana National Reserve on transects that covered approximately 20 ha of forest near existing trails between km 24 and km 28 of the Iquitos–Nauta highway. Root and leaf samples were collected in 2016 and 2021, respectively. We sampled 31 species (see Figure 1C). From each species, young and mature tissues were collected in the field from plants from permanent plots (see [34]), fixed in silica gel, and kept in a freezer (−20 °C) until analysis. Details of the foliar chemical analysis are found in Salazar et al. (2018) [34]. For leaf material, between six and nine different individuals per species were analyzed (more samples were used for species with higher intraspecific variation). For root material, between 3 and 19 samples per species were used for the chemical analysis (see Table S1 for detailed sample peer tissue counts).

### 2.2. Chemical Analysis

#### 2.2.1. Roots

Given the differences in the tissues and sample availability, it was not possible to use the same extraction approach for both leaves and roots. Therefore, here, we used a scale-down approach for roots designed to yield similar average peak areas with smaller amounts of dry plant material. First, we selected roots from each individual that had a maximum of 1 mm diameter in size. Fine roots were ground using a Bead Mill (ver. 24) and individual samples were weighed at 20 mg. We extracted tissues with 150 µL volume of 1:1 dichloromethane:ethanol (with 0.012 mg/mL caryophyllene oxide as internal standard). Samples were shaken in the bead mill for extraction, then pulse-centrifuged. The resulting tissue-solvent mixture was transferred into Costar Spin-X filter tubes (0.45 µm cellulose acetate) and centrifuged for filtration. Finally, filtrates were moved to glass autosampler vials for analysis with GCMS (Agilent 7890 Series, Agilent Technologies, Santa Clara, CA, USA). GCMS analysis consisted of the injection of 1.8 µL of plant extract for each sample into a split/splitless glass wool liner (Restek, Bellefonte, PA, USA). The inlet was heated to 270 °C in splitless mode. The oven method was as follows: 75 °C; ramp 5 °C/min to 200 °C; ramp 8 °C/min to 275; hold for 15 min; ramp 15 °C/min to 290 °C. The following mass spectrometry conditions were used: electron ionization source with positive ionization, 70 eV, scanning range 50–600 amu, and sampling rate = 50 Hz.

#### 2.2.2. Leaves

Details on the chemical analysis of leaf material are included in Salazar et al., 2018. In short, between 6 and 9 different individuals per species were analyzed. For each sample, 20 mg of the sample was extracted using 150 microliters of 1:1 dichloromethane:ethanol and 0.075 mg/L of Piperine as an internal standard. Extraction was performed in Costar Spin-X filter tubes (0.45 µm cellulose acetate, Sigma-Aldrich, St. Louis, MO, USA) and centrifuged for filtration. Finally, flow-through was transvased to autosampler vials. We injected 2.5 microliters of plant extract into a 4.0 mm ID Low Pressure Drop Precision Inlet liner (with glass wool; Restek, Bellefonte, PA, USA). The inlet was kept at a constant temperature of 275 °C. We used split injection with a 6:4 ratio. The oven was programmed as follows: 85 °C, hold for 2 min; ramp 1: 10 °C/min; 155 °C, hold for 1 min; ramp 2: 6 °C/min; 260 °C, hold for 1 min; ramp 3: 2 °C/min; 300 °C hold for 14 min (total run time 60 min). No column guard was used. MS conditions were as follows: EI source with positive ionization, 70 eV, scanning range 40–550 amu, and rate = 1 scan/ms. To assess carryover and retention time shifts, we injected a sample of solvent containing two internal standards (Piperine and Limonine) between every sample. These “Blank” samples were analyzed under identical chromatographic conditions described above.

Note on the analytical approach: Although our GCMS can detect only a subset of the total phytochemical metabolome likely present in a plant tissue, the results of previous studies using combinations of complementary hyphenated approaches suggest that GCMS can detect a significant percentage of the specialized compounds found in *Protium* [34]. Moreover, by using the same analytical method across all samples and tissues, we can systematically compare the chemical composition of leaves and roots across the same group of GCMS-detectable compounds. It is important to underline that although GCMS can be used to analyzed volatile and semivolatile compounds, not all compounds that are generally considered “volatile” are detectable via GCMS, and some compounds not generally considered “volatile” are indeed detectable via GCMS. Therefore, for this paper, we use the term “volatile” as an umbrella term that encompass compounds that are detectable via simple GCMS approaches.

### 2.3. Statistical Methods

GCMS data were preprocessed in MzMine (ver. 3.4) to perform peak detection and deconvolution. Compounds were identified using the NIST library (2017). Comparing the abundance of chemical compounds across samples and species is a complex challenge: different samples of the same species might present very different concentrations of the same specialized compounds based on genetic, developmental, and environmental factors including induction from natural enemies. And when examining chemistry across species, compounds might also differ in the intensity of their responses to chromatographic detection methods. Furthermore, it is hard to assess the biological value of concentration without some understanding of the biological activity of the compounds measured. Thus, to make meaningful contrasts between root and leaf specialized metabolites we (a) use relative peak areas for all compounds and (b) center root and leaf chemical data such that the average relative peak area for roots is comparable to the average relative peak area for leaf specialized metabolites. Although this approach does minimize the absolute chemical differences between above- and belowground plant tissues, it is a conservative approach that allows for easier interpretation of results when comparing diversity, composition, and phylogenetic patterns of the presence/absence and structural similarity of plant metabolites.

The final datasets yielded 403 compounds found in *Protium* leaves and 1040 in roots. To compare the divergence in the structural diversity of specialized metabolites between leaves and roots, we also created a data subset with the “top 50” compounds with the highest peak abundances (summed across all species). The goal of the “top 50” subset was to be able to compare the differences in chemical composition and structural diversity independent of the large differences in chemical richness between the two tissues.

To assess structural differences between specialized metabolites, we determined Spec2Vec distances between all *Protium* compounds. Spec2Vec is a method for quantifying the structural similarities between molecules based on their fragmentation spectra. Built upon a natural language processing algorithm, Spec2Vec offers an alternative to cosine-based spectral similarity scores, which are most effective at calculating structural “distances” between very similar molecules [35]. Given our focus on a broad range of metabolites across species, we elected to use Spec2Vec scoring over cosine-based methods to improve estimates of structural distances between molecules and allow us to compare molecules with no shared fragmentation features.

To obtain structural distances between compounds, we first processed the chromatographic data via Mzmine (3.4) and created annotated compound libraries (MGF files) for both root and leaf data. Then, we used Python 3.8 in CONDA with the Matchms package (v. 0.26.4) [36]. Here, we used the MoNA public database (MassBank of North America) and the combine compounds library for roots and leaves to create a relational model necessary to calculate structural similarities between compounds with little to no overlap in fragmentation features. We removed all compound mass spectra with 10 or less fragments per spectra as compounds with very few fragments in their mass spectra might have shown artificially low structural differences. After this, we used Spec2Vec to calculate distance matrices for our metabolite datasets (Python code is included in the Supplemental Materials). For all analyses of structural diversity (i.e., mean pairwise distances and beta diversity), root and leaf volatile metabolomes were filtered to include only compounds present in both the peak abundance dataset and Spec2Vec distance matrix: 140 compounds for roots and 449 for leaves.

Mean pairwise structural distances between species were calculated using the function ses.mpd from the R package picante (v. 1.6.0) [37]. For comparisons of compound richness and structure between roots and leaves, we used analysis of variance (function: aov; package: stats) [38]. For regression analysis, e.g., between the mean pairwise distances of compounds and total richness in roots, we used generalized linear models (glm; stats package v. 3.5). To test for correlations of values in distance matrices, e.g., phylogenetic versus structural distances, we turned to Mantel tests (mantel; vegan package; v. 2.5.2) [39]. Phylogenetic signals of individual specialized metabolites were calculated using multiPhylosignal from the picante package [37].

## 3. Results

Leaf volatile metabolites were dominated primarily by terpenoid compounds [34] while the most common volatile compounds identified in roots were phenolic and fatty acids. Roots were more “chemically rich” than leaves, having higher numbers of compounds (F = 130.9, DF = 60, *p* < 0.001; Figure 1A). On average, species expressed 116 detectable compounds in their leaves but 193 in their roots (median: 104 vs. 195). However, these chemically rich root volatile metabolomes were less structurally diverse than those of leaves (full structural dataset: F = 347.8, DF = 60, *p* < 0.001; top 50: F = 168, DF = 60, *p* < 0.001; Figure 1B and Table S1), and incorporating structural data reduced estimates of compound turnover between species in beta diversity calculations (Table S2). We saw no significant correlation between leaf and root compound richness (Est = −0.10, t = −0.563, *p* = 0.58; full structural dataset), even after ranking species to account for overall richness differences in chemical richness (Figure 1C). See the Supplemental Materials for a list of the putative identifications of the “top 50” metabolites from leaf and root tissues across our 31 *Protium* species.

**Figure 2:**
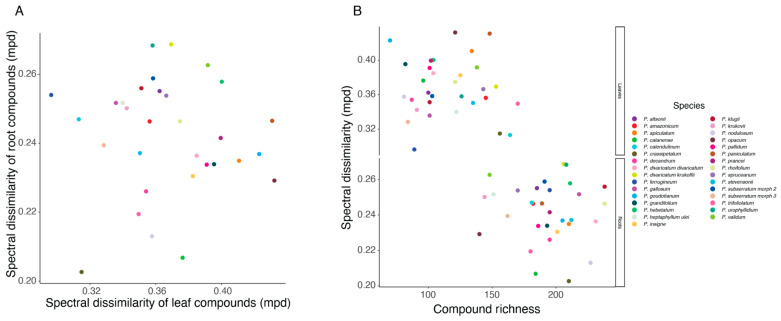
(**A**) The structural diversity of the “top 50” metabolites found in leaves and roots was not correlated across *Protium* species. The species metabolomic richness (full structural dataset) did not show any correlation with the species structural diversity values of compounds (**B**).

The structural diversity of compounds in leaves also did not correlate with the structural diversity in roots (full structural datasets: Est = −0.067, t = −0.179, *p* = 0.86; top 50 compounds: Est = −0.296, t = −1.02, *p* = 0.316; Figure 2A). And in both leaves and roots, there was no significant correlation between the structural diversity and richness of species’ specialized metabolites (full structural datasets: Est = −0.0001, t = −0.466, *p* = 0.64; top 50 compounds: Est = −0.00005, t = −0.41, *p* = 0.683; Figure 2B).

**Figure 3:**
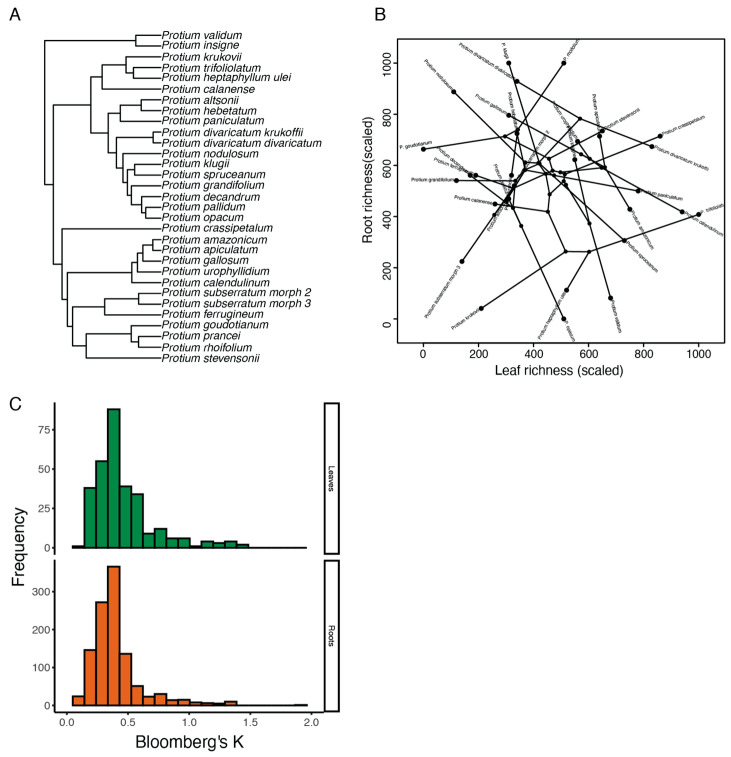
(**A**) We sampled roots and leaves from 31 species in the *Protium* phylogeny (including two well-defined subspecies of *P. subserratum*). (**B**) There was no correlation between root and leaf compound richness across the phylogeny. Shown here is the phylogeny of sampled *Protium* species projected into two-dimensional chemical trait space (root and leaf chemical richness). Chemical richness for leaves and roots has been scaled from 1 to 1000 to facilitate interpretation and visualization. The majority of individual compounds expressed moderately low values of Bloomberg’s K in both tissues, indicating high evolutionary lability of most metabolites (**C**).

In both roots and leaves, the vast majority of compounds were expressed by a handful of species; compounds were only shared by an average of six species in roots (median = 2) and nine species in leaves (median = 6; Table S3). We found that species’ phylogenetic relatedness (Figure 3A) only helped predict the structural similarity of metabolites in roots, with a weak positive correlation between phylogenetic and average structural distance (Figure 3C, Mantel r = 0.17, *p* = 0.02). There was no overall effect of common ancestry on the structural similarity of leaf specialized metabolites (Mantel r = 0.04, *p* = 0.30). When only taking into account compound identity and abundance, and not structural similarity, the phylogenetic signal of the vast majority of compounds was moderately low (Bloomberg’s K, Figure 3C).

## 4. Discussion

Here, we found strong evidence that *Protium* root and leaf specialized chemistries show distinct evolutionary trajectories, likely mediated by differences in their natural enemy communities, ecological contexts, and evolutionary constrains. Despite having significantly higher levels of chemical richness, small-molecular-weight volatile compounds in roots are more structurally similar across species than those in leaves. Closely related *Protium* species are also more likely to express structurally similar compounds in their roots. Contrastingly, *Protium* species had lower chemical richness but very high chemical structural diversity in their leaves. There was also no sign that phylogeny could predict foliar structural chemical diversity. Our work echoes findings in other taxa where leaves have shown strong signs of rapid divergence in chemistry among close relatives [5] and suggests that selection pressure for divergent chemistry may be much reduced belowground compared to aboveground.

The specialized chemistry of *Protium* roots and leaves was highly species-specific, with the vast majority of compounds only being expressed by a handful of species. Our findings match those of other studies on leaf metabolomes at the community scale [40]. Interestingly, species that showed high similarity in their leaf chemistry (based on structural distances between compounds) did not show high similarity in root chemistry. Ours was one of the first studies to examine phytochemical diversity across plant tissues through the lens of structural diversity. Our results showing greater compound richness in roots compared to leaves counter some recent work in subtropical trees that found similar metabolomic richness values above- and belowground using LC-MS, although root exudates were found to be exceptionally rich [24].

The richness, or number of compounds that a plant produces, has long been a key measure of chemical diversity [41] and has been shown to be a strong predictor of the diversity of associations between plants and herbivores in *Protium* [42]. It is yet to be determined whether the differences in chemical richness between above- and belowground tissues in *Protium* mirror the diversity and richness differences in plant–enemy interactions. Nevertheless, in tropical environments, fungal pathogen species are likely to vastly outnumber insect species [43]. Measurements of compound structural differences, while still in their nascency [44,45], can significantly improve our ability to link measures of chemical diversity and richness to biological processes, as one might do by incorporating phylogenetic relatedness in assessments of community-level species richness. As could be expected, we found that incorporating structural information about compounds generally tempered our estimates of beta diversity or differences between species’ chemical compositions. There was no correlation between chemical richness and structural diversity across species and tissue types, suggesting a broad decoupling of these two phenotypic measures at an evolutionary scale. The lower structural diversity found belowground might reflect evolutionary and ecological constrains uniquely found in root–fungus interactions. While evolutionary processes in leaves can “explore” a larger phytochemical universe, the evolution of chemical defenses in root systems is likely more constrained to prevent the disruption of interactions between plants, mycorrhiza, and symbiotic bacteria. However, more studies are necessary to link the conservation of specific chemical structural motifs to these belowground constrains.

Across the GCMS detectable metabolome, we found a similar distribution of phylogenetic signals of compounds in roots and leaves, suggesting that most chemical traits are evolutionarily labile both above- and belowground. Yet, closely related *Protium* species showed more structural similarity than distantly related species. We suggest that this could reflect stronger pressure from aboveground natural enemies (e.g., herbivores) towards the rapid divergence of leaf metabolomes. Similarly, it seems to suggest that sympatric *Protium* species are not under a strong selective pressure to disverge chemically belowground. Recent work by the Peay lab has shown that fungal and oomycete communities vary widely across *Protium* species with high levels of turnover (Alvarez and Venturini et al. in prep), likely reducing selection pressure for metabolomic divergence.

Given that root compounds can act as a signal of infection to beneficial microbes [46], the structural similarity of compounds may not only be a sign of relaxed selection pressure due to lower levels of specialization by underground enemies compared to those aboveground but also aid in the attraction of generalist mutualists. Yet, disentangling these two components and their selective outcomes can only be carried out through detailed experimental work.

There is much more to explore in the evolutionary trajectories of specialized metabolites across plant tissues: in particular, linking phylogenetic patterns of diversity and composition in particular compound classes to interactions with enemies or mutualists. While it may be expected that different metabolites will dominate different plant tissues, our results suggest that we should also expect the evolution of plant specialized metabolism to reflect differences in the strength and specificity of interactions with other organisms above- versus belowground.

## Data Availability

The dataset is available on request from the authors.

## Supplementary Materials

The following supporting information can be downloaded at: https://www.mdpi.com/article/10.3390/plants14020225/s1, Table S1: List of samples per species per tissue analyzed; Table S2: List of the “Top 50” metabolites produced in root tissued across all our *Protium* focus species; Table S3: List of the “Top 50” metabolites produced in leaf tissued across all our *Protium* focus species.
