# Supplementary material for: Evolutionary Trajectories of Shoots vs. Roots: Plant Volatile Metabolomes Are Richer but Less Structurally Diverse Belowground in the Tropical Tree Genus Protium
Source: Plants (Basel). 2025 Jan 15;14(2):225. doi: 10.3390/plants14020225 (PMC11769111; doi:10.3390/plants14020225)
Supplement: Supplementary file 1 [file plants-14-00225-s001.zip › plants-3389905-supplementary.pdf]

Evolutionary Trajectories of Shoots vs. Roots: Plant Volatile Metabolomes Are Richer but Less Structurally Diverse Belowground in the Tropical Tree Genus *Protium*

Katherine D. Holmes [1,2,*], Paul V.A. Fine [3], Italo Mesones [3], Julieta Alvarez-Manjarrez [4], Andressa M. Venturini [5],
Kabir G. Peay [5] and Diego Salazar [1,2]

[1] Department of Biological Sciences, Binghamton University, Binghamton, NY, USA. Location; dsalazaramor@binghamton.edu
[2] Biology Department, Florida International University, Miami, Florida, USA. Location;
[3] Department of Integrative Biology, University of California Berkeley, Berkeley, California, USA Location;
paulfine@berkeley.edu (P.F.); italomesones@berkeley.edu (I.M.)
[4] Universidad Nacional Autónoma de México, Mexico D.F., Mexico. Location; julieta.alvarez@ib.unam.mx
[5] Stanford University, Stanford, California, USA. Location; aventuri@stanford.edu (A.M.V.); kpeay@stanford.edu (K.P.)
* Correspondence: kholmes4@binghamton.edu

Supplementary material:

Table S1: List of samples per species per tissue analyzed for this study. Defenses in sample number are a function of sample availability, rareness in the field, amount of tissue available at time of collection and at time of analysis.

**Species**	**Number of samples for leaves**	**Number of samples for Roots**
Protium altsonii	4	3
Protium amazonicum	3	19
Protium apiculatum	4	4
Protium calanense	5	9
Protium calendulinum	6	5
Protium crassipetalum	3	15
Protium decandrum	4	9
Protium divaricatum divaricatum	5	11
Protium divaricatum krukoffii	5	14
Protium ferrugineum	6	10
Protium gallosum	5	11
Protium goudotianum	7	8
Protium grandifolium	4	4
Protium hebetatum	7	7
Protium heptaphyllum ulei	4	12
Protium insigne	3	15
Protium klugii	5	17
Protium krukovii	4	4
Protium nodulosum	6	10
Protium opacum	3	16
Protium pallidum	8	11
Protium paniculatum	5	11
Protium prancei	7	9
Protium rhoifolium	4	3
Protium spruceanum	9	4
Protium stevensonii	4	7
Protium subserratum morph 2	10	12
Protium subserratum morph 3	8	15
Protium trifoliolatum	4	4
Protium urophyllidium	5	5
Protium validum	5	17

Table S2: List of the "Top 50" metabolites produced in root tissued across all our *Protium* focus species. These putative identifications are based on matches on the NIST and MoMA databases. If a matches from both data bases disagreed, we used the NIST database match as this database is better curated. Please note these all our focus species are non-model organisms and therefore, some of these matches are likely include some degree of uncertainty.

**"Top 50" Root metabolites detected via GCMS**
(6R,7R)-Bisabolone
(E)-2,6-Dimethoxy-4-(prop-1-en-1-yl)phenol
Globulol
1,3,7-Octatriene, 3,7-dimethyl-
Friedelan-3-one
2-Hydroxy-4-methoxy-alpha-(p-methoxybenzylidene)acetophenone
2-Propanol, 1-(2-butoxy-1-methylethoxy)-
2,6-Dimethyl-2-trans-6-octadiene
5-Hydroxy-4,7-dimethoxyflavanone
9-Octadecenamide, (Z)-
Acetophenone
Acetophenone, 2,4-dimethoxy-3-methyl-
Amyrin
Benzaldehyde, 3-hydroxy-4-methoxy-
Benzene, 1-methyl-4-(1-methylethenyl)-
Benzoic acid
Bisabolol
Butylsyringone
Calacorene
Cholesta-4,6-dien-3-ol, (3)-
Clionasterol acetate
cis-Calamenene
Cyclohexene, 4-ethenyl-4-methyl-3-(1-methylethenyl)-1-(1-methylethyl)-, (3R-trans)-
D-Limonene
Dimethyldaidzin
endo-Borneol
Epicurzerenone
Geranyl ethyl ether 1
Hexadecane
Hexadecanoic acid, 2-hydroxy-1-(hydroxymethyl)ethyl ester
m-Cymen-8-ol
Myrcene
n-Hexadecanoic acid
Neointermedeol
Nonanamide
o-Cymene
Octadecanamide
Octadecanoic acid
Octadecanoic acid, 2,3-dihydroxypropyl ester
p-Cymene-2,5-diol
Phenol, 4-ethenyl-2,6-dimethoxy-
Phenol, 4-methoxy-3-methyl-
S-Verbenone
Sitosterol
Squalene
syringic acid
Syringylacetone
Thymol
trans-Calamenene
β-Santalene

Table S3: List of the "Top 50" metabolites produced in leaf tissued across all our *Protium* focus species. These putative identifications are based on matches on the NIST and MoMA databases. If a matches from both data bases disagreed, we used the NIST database match as this database is better curated. Please note these all our focus species are non-model organisms and therefore, some of these matches are likely include some degree of uncertainty. This list includes 8 unknowns. If good matches are not found in the databases, compounds are classified as unknowns and given the UNK label plus a number. Fragmentation data on these UNK compounds is available upon request.

"Top 50" Leaf metabolites detected via GCMS
1,2-15,16-Diepoxyhexadecane
14-Methyl-5-ergosta-8,24(28)-dien-3-ol
24-Norursa-3,9(11),12-triene
28-Norolean-17-en-3-ol
3,4-Divanillyltetrahydrofuran
9,12,15-Octadecatrienoic acid, (Z,Z,Z)-
Amyrin
Amyrone
Apiol
Bicyclo[3.1.1]hept-2-ene, 3,6,6-trimethyl-
Bicyclogermacrene
Bisabolene
Caryophyllene
Copaene
Cubebene
Cycloartenol
D-Limonene
Ethyl 2-hydroxybenzyl sulfone
Germacrene D
Hexadecanoic acid, 2-hydroxy-1-(hydroxymethyl)ethyl ester
Humulene
Lanosterol
Lupeol
Methyl 6-cis,9-cis,11-trans-octadecatrienoate
n-Hexadecanoic acid
Neophytadiene
Nonadecane
Octadecanoic acid
Octadecanoic acid, 2,3-dihydroxypropyl ester
p-Cymene
pathulenol
Phellandrene
Phytol
Sitosterol
Squalene
Stigmasterol
Sylvatesmin
tau-Cadinol
Tocopherol
Tocospiro A
UNK23
UNK34
UNK36
UNK37
UNK38
UNK39
UNK40
UNK80
Vinyl decanoate
α-gurjunene
